# Economic, socio-emotional, and food security conditions during COVID-19 pandemic among caregivers of young adolescents aged 10–12 yrs in a semi-urban setting in Ghana

**DOI:** 10.21203/rs.3.rs-7215235/v1

**Published:** 2025-08-21

**Authors:** Mavis O. Mensah, Ebenezer Adjetey, Lois M.D. Aryee, Charles D. Arnold, Elizabeth L Prado, Paul D. Hastings, Amanda Guyer, Brietta M. Oaks, Helena Nti, Helena J. Bentil, Jonnatan Fajardo, Seth Adu-Afarwuah

**Affiliations:** University of Ghana; University of Ghana; University of Ghana; University of California-Davis; University of California-Davis; University of California-Davis; University of California-Davis; University of Rhode Island; University of Ghana; University of California-Davis; University of California-Davis; University of Ghana

**Keywords:** COVID-19, caregivers, young adolescents, economic, education, household food situation, Ghana

## Abstract

**Background:**

Few studies have described the consequences of the COVID − 19 pandemic among caregivers of young adolescents in sub-Saharan Africa. We aimed to explore the consequences of COVID-19 on economic, socio-emotional, and household food security conditions among caregivers of young adolescents in a semi-urban setting in the Eastern Region of Ghana.

**Methods:**

In this cross-sectional study, caregivers of young adolescents aged 10–12yrs in the Somanya-Kpong area were enrolled. These participants were part of the second follow-up of the iLiNS-DYAD Ghana trial. A questionnaire designed from the PhenX Toolkit COVID-19 Protocol and the Food Access and Food Security During COVID-19 Survey (Version 2.1) was used to collect data over 8 months starting January 2022. We used descriptive statistics to summarize data and McNemar Chi-square tests to compare percentages of agreement to statements of household food security conditions “*one year before”* versus “*since”* the pandemic outbreak.

**Results:**

Among 966 caregivers (94% females; 6% males), 89% reported decreased household income compared with the year before the pandemic. Although 72.5% of caregivers continued working during the pandemic, most said they had experienced a reduction in their work hours (72.6%), workload (78.8%) and salaries (63.4%). Many (65%) said their children engaged in educational activities when schools were closed, with 46% hiring private teachers. Caregivers most frequently cited financial concerns (83%) and negative impact on work (79%) as their greatest sources of stress because of COVID-19. Significantly more caregivers reported experiencing household food insecurity conditions “*since the outbreak*” compared to “*one year before the outbreak*” (55.4% vs 19.6%, P < 0.05).

**Conclusions:**

In this setting, COVID-19 had negative consequences on the economic, socio-emotional, and household food security conditions of caregivers and their young adolescents.

**Trial registration:**

Clinical trial number not applicable.

## Background

The Coronavirus Disease 2019 (COVID-19) pandemic severely disrupted public health, economic, and social systems worldwide [1, 2, 3]. As of 31 December 2023, COVID-19 had been confirmed in over 773 million people worldwide, with over 7 million deaths and many more experiencing adverse longer-term health consequences after being infected [4, 5].

In Ghana, the first official cases of COVID-19 were reported on 12 March 2020 [6]. The Government of Ghana quickly implemented containment measures, including border closures, partial lockdowns in major urban cities, bans on all public gatherings (e.g., conferences, workshops, funerals, festivals, political rallies, churches, mosques etc.), and closure of all educational institutions lasting for 10 months from March 2020 to January 2021 [6, 7, 8, 9]. COVID-19 protocols such as wearing face masks, handwashing, use of sanitizer, and social distancing, were strictly enforced and/or adhered to in all public places [1, 10].

The government declared on 28 May 2023 that the pandemic was no longer of significant threat [11]. By this time, countless livelihoods were altered, and the economic burden and hardships many experienced due to the pandemic had been immense [12, 13, 14, 15]. For example, during the first two months of the pandemic, approximately 42,000 Ghanaians lost their jobs [12]. There was an increase in unemployment rate [12, 13], a reduction in income [14], a reduction in per capita household consumption of food and non-food goods [14, 16] and increased psychological distress [1]. Education was also significantly impacted. With the closure of schools for 10 months, nearly 9.2 million Ghanaian students in basic school (i.e., kindergarten, primary, and junior and senior high schools) and 0.5 million in tertiary institutions (i.e., college, university) had been affected. Although virtual platforms were largely launched to facilitate learning among school children and students, these were not accessible to all students [15]. These consequences of the pandemic disproportionately affected vulnerable populations, including children and their families, the poor, the elderly, and people living in rural areas [8, 9, 17].

The socioeconomic, emotional, and food security disruptions caused by the COVID-19 pandemic may be widespread, but the available evidence for people in various vulnerable settings in Africa is limited. A recent scoping review identified only 22 studies of the impacts of the pandemic on the well-being of families in Africa [18], with only a fraction of these considering adolescence specifically. In Ghana, the impact of the pandemic on households has predominantly been examined through nationwide statistics, which may mask substantial sub-national variations [7, 9, 14, 19]. Additionally, most of these studies focused on areas included in the government’s COVID-19 lockdown [20], which were predominantly urban. Hence, the impacts of the pandemic on the wellbeing of more than 40% of Ghana’s families [21] remain unknown.

Examining the impact of the COVID-19 pandemic among households in semi-urban settings in Ghana is of considerable importance for understanding the various challenges these communities faced. Such areas at the interface of urban and rural zones are increasingly common in Ghana, affecting the lifestyles of localities that previously subsisted primarily through farming [22]. Typically, such communities in Ghana have limited access to social amenities and employment opportunities, making it likely that many caregivers of children in these areas particularly vulnerable during the COVID-19 pandemic.

This research leveraged an existing cohort of young adolescents enrolled in the second follow-up study of the International Lipid-Based Nutrient Supplements (iLiNS) DYAD Ghana trial in the Somanya-Kpong area [23] to focus on the caregivers of these young adolescents. The area is a semi-urban setting located approximately 70 km north of Accra [24]. It was likely that the impacts of the pandemic in this area reflected patterns in similar settings in Ghana, particularly in terms of economic stability, socio-emotional well-being, and food security. The pre-existing relationship between the families of these young adolescents and our research team provided an opportunity to engage with the caregivers who were amenable to discuss how the pandemic affected their livelihoods and families.

Considering these circumstances, the present study aimed to explore the consequences of COVID-19 on the economic, socio-emotional, and household food security conditions of caregivers and their young adolescents living in a semi-urban setting in Ghana.

## Methods

### Study design, participants, and site

This cross-sectional study was conducted in the Somanya-Kpong area in the Yilo Krobo and Lower Manya Krobo districts in the Eastern Region of Ghana, about 70 km north of Accra, the national capital. Participants were the primary caregivers of young adolescents aged 10–12 years who were taking part in the second follow-up study of the iLiNS DYAD Ghana randomized trial [23].

Most residents of the area were subsistence farmers or petty traders and were middle class, by Ghanaian living standards [24]. Their staple foods consisted of maize, cassava, rice, fish, and leafy vegetables. The area was not included in the 3-week COVID-19 total lockdown of parts of the country (i.e., the Greater Accra Metropolitan Area and the Greater Kumasi Metropolitan Area) from 30 March 2020 [25, 26], but experienced other government-instituted COVID-19 prevention measures, including closure of basic schools [27] (from March 2020 until after January 2021), a ban on public gatherings [28] (e.g., sporting, funerals, festivals, and church activities from March to August 2020), fumigation of markets and schools (April to July 2020), and enforcement of social distancing and wearing of face masks throughout the pandemic [29]. The Government of Ghana also instituted measures across the country, including distance learning via radio, television, and online platforms to mitigate academic disruptions [30]; it was unlikely these measures were effective in the study area due to poor access to Information and Communication Technology infrastructure.

### Enrolment and data collection procedures

Participant’s sociodemographic information were collected between December 2020 and December 2021 as part of the follow-up study they were participating in. From 28 January 2022 to 23 September 2022, field workers re-contacted the caregivers using the last-known telephone numbers and/or home addresses. We obtained written informed consent and assent from the caregivers and adolescents for the present study. Subsequently, field workers administered a COVID-19 related questionnaire, which we designed from the PhenX Toolkit COVID-19 Protocols (https://www.phenxtoolkit.org/protocols) [31] and the Food Access and Food Security During COVID-19 Survey Version 2.1 [32]. Specific questions were selected to cover relevant domains including household income, finances, and employment (n = 13), education (n = 13), emotional and social behavior (n = 14), and food access and security (n = 2) (see Supplemental Material 1). The questions were administered in three local languages (*Twi*, *Ewe*, and *Ga-*A*dangme*), and we asked participants to focus on *one year before the COVID-19 outbreak* as against the *period since the COVID-19 outbreak*.

The COVID-19 questionnaire was programmed on tablets using the SurveyCTO software. SurveyCTO has in-built quality checks to minimize the entry of implausible values. Seven field workers were trained to administer the questionnaire, and a standard operating procedure manual was developed to ensure consistency in administering the questionnaire. The questionnaire was piloted among 20 caregivers in the study area who were not part of the iLiNS-DYAD follow-up study to assess feasibility, duration, and adaptation in the local context.

### Outcome variables

First, we inquired about household and caregiver’s income situation during the COVID-19 pandemic, including (i) “*Has your household income changed since the COVID-19 outbreak?”*, (ii) *“Did you lose any jobs during the COVID-19 outbreak?”* and (iii) “*Did you experience significant changes in your job because of or during the COVID-19 outbreak?”*.

Second, we inquired about children’s learning conditions during the pandemic, including (i) “*During the time of school closure, were school children in this household doing educational activities at home?”*, (ii)*“What were the sources of educational or learning activities for children in this household during the school closures?*”; (iii) *“Who assisted the children with educational activities?”*; and (iv) “*How has the outbreak affected your childcare while school was suspended?*”

Third, to collect caregiver’s report of changes in their children’s and their own emotional behavior and social connectedness before versus during the COVID-19 pandemic, we used (i) a Likert scale from 1 (much less often) to 5 (much more often) to measure the following questions a. *“Indicate the extent to which young children in this household felt irritated, afraid, and sad before the COVID-19 and since the COVID-19 outbreak (during the school closure)”*,b. *“Indicate the extent to you felt irritated, afraid, and sad before the COVID-19 and since the COVID-19 outbreak (during the school closure)”* and (ii) a Likert scale from 1 (less socially connected) to 6 (much more socially connected) to measure the question, “*Indicate the extent to which the child seems socially connected to other people and his environment before the COVID-19 outbreak and during the COVID 19 (especially during the school closures)*”. In addition, we used a Likert scale from 1 (extremely negative) to 7 (extremely positive) to measure the question, *“indicate the extent to which you view the COVID-19 outbreak as having either a positive or negative impact on your life”*. We inquired about caregivers’ stress during the pandemic including a. “*What have been your greatest sources of stress from the COVID-19 outbreak?*” and b. *“What have you done to cope with your stress related to the COVID-19 outbreak?”*.

Finally, caregivers reported household food security conditions (i) on a Likert scale from 1 (not at all worried) to 6 (extremely worried), measuring the question *“On a scale from 1 (not at all worried) to 6 (extremely worried), what is your level of worry for your household about the following as it relates to COVID-19”*, and (ii) agreement (“never true”, “sometimes true” and “often true”) with statements about their household’s food situation in the last year before the COVID-19 and since the COVID-19 outbreak

## Data analysis

A statistical analysis plan was prepared prior to the data analysis. The data were analyzed using R version 4.2.1 supported by MS Excel 2016. Descriptive statistics were used to summarize participants’ background characteristics and the outcome variables.

Due to low frequency of responses for certain categories across many of the Likert scale variables and to ensure uniformity and consistency in the presentation of results, we dichotomized the Likert scale ratings. The 5-point Likert scale ratings of the extent to which caregivers and children (by caregivers’ reports) felt irritated, afraid, anxious, and sad before and during the COVID-19 pandemic were dichotomized as “often” (if caregivers responded, “much more often”, “more often” or “somewhat often”) or “not often” (if caregivers responded, “much less often” and “less often” responses). We calculated the percentage of caregivers and children who reportedly “often” felt irritated, afraid, anxious, or sad before and during the COVID-19 pandemic.

The 6-point Likert scale ratings of the extent to which children (by caregivers’ reports) were socially connected to other people and their environment before and during the COVID-19 pandemic were dichotomized as “socially connected” (if caregivers reported “much more socially connected”, “more socially connected” or “slightly more socially connected”) and “not socially connected” (if caregivers reported “much less socially connected”, “less socially connected” and “slightly less socially connected”). We calculated the percentage of children who reportedly were “socially connected” to other people in their environment before and during the COVID-19 pandemic.

We calculated the mean (SD) score of the 6-point Likert scale ratings of caregivers’ level of worry about food situation during COVID-19 and dichotomized the 3-point Likert scale responses of caregiver’s agreement with statements about their household food situation to “yes” (for “sometimes true” and “often true”) or “no” (for “never true”). Finally, we used McNemar Chi-square tests to compare percentages of caregivers with agreement (“yes”) to the statements about their household food situation “*one year before the outbreak*” versus “*since the outbreak*”. A significance level of p < 0.05 was used.

## Results

The background characteristics of the caregivers interviewed are shown in Table 1. Of the 966 caregivers, 93.6% were females. Most were self-employed mainly as traders and shop owners before COVID-19 (83%), married or in an informal union (81%), and had education up to Junior High School (73%). The household members of respondents who were in school at the time of data collection (n = 2970) consisted predominantly of those in primary (56.6%) and Junior High (16.4%) schools.

### Household income, caregivers’ working situation, and children’s learning conditions (by caregivers’ report) during the COVID-19 pandemic

Table 2 summarizes the reported changes in household income, caregivers’ working situation, and children’s learning conditions *(by caregivers’ report)* during the COVID-19 pandemic. Most caregivers (88.5%) reported less household income due to the pandemic; 27.5% reportedly lost their jobs because of the pandemic. Changes in caregivers’ working conditions most frequently reported during the COVID-19 pandemic were decreased workload (reported by 79% of caregivers) and salary or wage cut (reported by 63% of caregivers). By contrast, increased workload and increased salary or wage were reported by only 2–6% of caregivers.

Table 2 also shows that during school closures due to the pandemic, 64.7% of caregivers said their children engaged in educational activities at home. The sources of educational or learning activities most frequently reported by caregivers were private teachers (46.2% of caregivers); educational TV programs (32.6%); and sessions, meetings and/or assignments provided by the schoolteacher (26.1%). The most frequent helpers with children’s educational activities were caregivers (65.8%), followed by the children themselves (47.0%), then other household members (26.0%) such as older siblings. Regarding whether the outbreak had affected caregivers ‘childcare while schools were suspended, the vast majority (81.8%) of caregivers reported no impact, except for a relatively small percentage who or whose partners reportedly changed their work schedule to care for their children (14.4%), had difficulty arranging for childcare (2.7%), or had to pay more for childcare (0.5%). Caregivers also reported that a greater percentage of their children were socially connected to other people and their environment before the pandemic (91.9%) versus during the pandemic (44.2%).

### Changes in emotional behavior of caregivers and children

**Figure 1:**
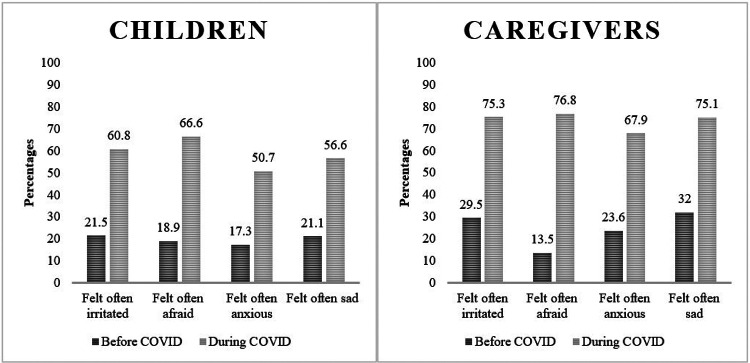
Percentage of children and caregivers feelings before and during the COVID-19 pandemic, by caregiver report.

*During* versus *before* the pandemic, greater percentages of children were reportedly “often irritated” (60.8% versus 21.5%), “often afraid” (66.6% versus 18.9%), “often anxious (50.7% versus 17.3%) and “often sad” (56.6% versus 21.1%) (Fig. 1). Likewise, greater percentages of caregivers were reportedly “often irritated” (75.3% versus 29.5%), “often afraid” (76.8% versus 13.5%), “often anxious (67.9% versus 23.6%) and “often sad” (75.1% versus 32%) *during* the pandemic compared with *before* the pandemic (Fig. 1).

### Caregivers’ perceived impact of COVID-19 and coping strategies

As shown in Table 3, most (89.9%) caregivers perceived that the impact of COVID-19 on their lives was “extremely negative” (40.7%), “moderately negative” (30.2%) or “somewhat negative” (19.0%), compared with those who reported “no” (6.1%) or “slightly positive”, “moderately positive” or “extremely positive” (4.0%) impact. The most frequently reported sources of stress due to COVID-19 were financial concerns (83.0% of participants) and impact on work (79.0%), access to food (47.8%), impact on child (47.1%), health concerns (27.2%), and impact on family members (21.0%). Other less frequently cited concerns were impact on the community (1.9%) and access to medical care; only 5.5% of caregivers were reportedly not stressed about the COVID-19 outbreak.

The coping strategies most frequently cited by caregivers for dealing with the stress related to COVID-19 were “meditation and/ or mindfulness practices” (81.4%), “talking with friends and family through phone, text or video” (46.0%), “increased television or other ‘screen time’ activities such as video games and social media (43.5%) and “engaging in family activities (11.0%).

### Caregivers’ response on indicators of household food insecurity

Table 4 shows that the mean (SD) Likert scale scores were high for several items of the household food conditions during the pandemic, including “household will not have enough food at home and cannot go out due to quarantine or illness” (5.0 (1.2)); “food will be more expensive for my household” (4.8 (1.4)); “my household will lose so much income that they cannot afford enough food” (4.7 (1.4)); “there will not be enough food in the marketplace (4.0 (1.7); and “the country will not have enough food to feed everyone (4.0 (1.7)).

For each of five statements of the household food situation, indicative of household food insecurity, the percentage of caregivers expressing agreement was significantly (p < 0.05) lower for *“one year before outbreak”* compared with *“since outbreak”* of COVID-19. These statements included *“food the household bought did not last and there was no money to get more*” (19.6% for *one year before outbreak* vs 55.4% for *since outbreak*); “*household could not afford to eat balanced meals*” (17.1% vs 48.8%); “*household members ate less food than they felt they should because there was not enough money for food*” (17.3% vs 42.7%); “*adults in the household ever cut the size of meals or skipped meals because there was not enough money for food*” (15% vs 41.4%); and “adults in the household were ever hungry but did not eat because there was not enough money for food” (6.0% vs 18.4%).

## Discussion

Our findings reveal the negative consequences of COVID-19 among caregivers of young adolescents 10–12y of age in a semi-urban area in Ghana. Most of the caregivers interviewed reported a decrease in household income, with a few losing their jobs, while others experienced reduced workloads and salary cuts. During the school closures, young adolescents used various channels to learn; most caregivers engaged the services of private teachers for the children at home or allowed the children to watch educational TV programs. According to caregivers’ reports, higher percentages of caregivers and their young adolescents felt “often irritated”, “often afraid”, “often anxious”, and “often sad” during the COVID-19 than before the pandemic. Most caregivers perceived the pandemic negatively impacting their lives, often stressed by financial concerns, work, and poor access to food, and coped mainly by meditation, mindfulness practices, and communication with friends and family via phone or video. In our study, there also was a significant increase in the perceived household food situation since the COVID-19 outbreak compared to the year before, with these Ghanaian caregivers expressing high levels of concern about household food conditions.

The reported decline in household income in our sample during the COVID-19 pandemic was consistent with reports from other studies in Ghana [16, 20, 33]; these reports highlighted the fact that informal self-employed workers typically experienced a fall in their household income during the COVID-19. The activities of self-employed workers, especially petty traders, mostly require physical contact; such workers mainly rely on daily sales as earnings, and this was massively affected by the pandemic [13, 34]. Schotte et al. (2021) report that in Ghana, the earnings of self-employed women declined drastically during the pandemic, which may have further heightened the existing labor inequalities in the country [20]. Our findings corroborate those reported by Schotte et al. (2021). Although household incomes generally declined during the COVID-19 pandemic, most of the caregivers reportedly did not lose their jobs and continued to work during the pandemic. This may be because a majority of respondents were self-employed, and our study area had not been included in the partial lockdown that was instituted by the government of Ghana during the pandemic [27]. Durizzo et al. (2021) confirmed that in districts where there were no lockdowns, many workers in the informal self-employment sector continued to work and only few people lost their jobs [35].

The variety of learning channels used during school closures suggests that there is no “one size fits all” approach to learning. The high cost and poor connectivity of internet services [36, 37] was a major problem in the study area; thus, it was not surprising that few people engaged in e-learning. The schools in the study area were mostly public schools, hence were not active in providing educational activities for children during the school closures. As such, parents and other family members had to take up much of the burden of providing their children with educational and learning resources during the pandemic. However, parents/caregivers may not have had the requisite educational background and skill set or be well equipped to teach their children at home [16]. This could partly explain why many of the respondents reported hiring a home teacher. Children from less privileged households without adequate means to engage home teachers may have faced difficulties in keeping up with their studies, potentially impacting their academic performance after school returned [16].

The increased prevalence of reported negative socio-emotional behavior (irritated, afraid, anxious, and sad) among caregivers and their young adolescents during the COVID-19 warrant discussion. Similar findings were reported among Ghanaian women and children [38] and Canadian families [39] as a result of the COVID-19 pandemic. Closures of schools and lockdowns caused children and caregivers to be confined at home, spending less time interacting and socialising with friends or people outside of their households. This social isolation may have contributed to the increased stress, anxiety, and even depression due to prolonged confinement and lack of social support. Constant close quarters with household members without external social interactions could have put a strain on family relationships, potentially leading to increased conflicts and tension at home, including excessive yelling and punishments from parents and other older family members [39]. For caregivers, additional stress may have resulted from financial difficulties, poor access to food, and possible work- and childcare-related problems due to the pandemic.

It was not surprising that many participants reported being worried about their households’ food situation, which may likely have been the negative consequences of decreased household income and/or loss of employment because of the COVID-19 pandemic. During the lockdowns, demand for food appeared to exceed supply, thereby pushing food prices up [40], as many food markets were closed to enforce social distancing and/or allow for fumigation. As many of the respondent reportedly ate less frequently and had reduced variety and quality of food, those changes could have contributed to increased stress among the participants and their children [41].

Our study had several strengths. The caregivers’ participation in the ongoing iLiNS-DYAD follow-up study probably resulted in a high response rate from the participants. Participation in the follow-up study likely increased the caregivers’ comfort with sharing their life experiences during the pandemic, potentially enhancing the reliability of the data collected. The interview questionnaire was administered in person, which likely helped maintain the depth and quality of the data.

The study had a few limitations. First, we had only one round of data collection after the outbreak. Given the multiple waves of the pandemic, the changes we observed may have varied over time. We began data collection about 2 years after the outbreak of the pandemic, and the data collection took about 9 months to complete, by which time the reported COVID-19 cases had declined and many containment measures had been eased. It is possible that participants’ adaptations over time may have diluted the perceived impact of the pandemic. This time lag may have introduced some recall bias due to the self-reported nature of outcome measures. However, all data were collected while the pandemic was still active and therefore reflected the ongoing conditions and experiences of participants. In addition, to keep the survey from being burdensome for participants, we did not collect sociodemographic information that had been collected 1–2 years previously; hence, it is possible that family characteristics may have been somewhat different than described in the method section.

## Conclusion

In this setting, COVID-19 had negative consequences on the economic, socio-emotional, and household food security conditions of caregivers of young adolescents. Our study findings contribute to understanding the impact of the pandemic in Ghana. The findings are comparable with other studies and add to the existing literature on the multiple disruptions and impact of the pandemic on livelihoods. This can help inform public health responses in future pandemics. To extend the findings of our study, further investigation on the long-term effect of the COVID-19 pandemic and how individuals and households recovered post COVID-19 can be conducted.

## Figures and Tables

**Table 1:** Background characteristics of caregivers (n = 966)

Characteristics	n (%)
**Gender of caregivers**	
Female	904 (93.6)
Male	62 (6.4)
**Employment status of caregivers before COVID-19**	
Self-employed	802 (83.0)
Salaried worker	132 (13.7)
Unemployed	32 (3.3)
**Marital status of caregivers**	
Married	509 (53.1)
Informal/loose union	267 (27.9)
Separated	77 (8.1)
Widowed	54 (5.6)
Divorced	28 (2.9)
Single (never married or in an informal union)	23 (2.4)
**Level of education of caregivers**	
Primary school	213 (22.2)
Junior high school	487 (50.8)
Senior high school	95 (9.9)
None	90 (9.4)
Other training institutions	38 (4.0)
Tertiary	35 (3.7)
**Household members currently enrolled in school** ^1^	
Pre-school or day care	419 (14.1)
Primary school	1680 (56.6)
Junior high school	488 (16.4)
**Gender of caregivers**	
Senior high school	299 (10.1)
Tertiary	84 (2.8)

1 Multiple response questions

**Table 2:** Caregivers’ reported household income situation, and children’s learning conditions (n = 966)

Variable	n (%)
***Has your household income changed substantially since the COVID19 outbreak?***	
My household income is less	855 (88.5)
My household income is about the same	97 (10.0)
My household income is more	14 (1.5)
**Did you lose any job(s) during the covid-19 outbreak?**	
Continued working	661 (72.5)
Lost job	251 (27.5)
**Did you experience significant changes with your job?** ^1^	
Decrease in workload	652 (78.8)
Decrease in work hours	600 (72.6)
Salary or wage decreased	525 (63.4)
No significant change	62 (7.5)
Increase in work hours	56 (6.8)
Increase in workload	52 (6.3)
Salary or wage increased	20 (2.4)
**During the time of school closure, were school children in this household doing educational activities at home?**	
Yes	625 (64.7)
No	341 (35.3)
**What were the sources of educational or learning activities for child(ren) in this household during the school closures?** ^1^	
Private lessons (Hired teacher)	290 (46.2)
Watched educational TV programmes	205 (32.6)
Session/meetings/ Completed assignments provided by schoolteacher	164 (26.1)
E-learning	24 (3.8)
Used mobile learning apps	14 (2.2)
***Has your household income changed substantially since the COVID19 outbreak?***	
Listened to educational programmes on radio	11 (1.8)
Other (group studies, parents/siblings, learning from old notes)	218 (34.7)
**Who assisted with the educational activities?** ^1^	
Caregiver or spouse/partner	413 (65.8)
Child themselves	296 (47.0)
Other household members	166 (26.0)
The school	25 (4.0)
Other (neighbors, friends, hired teachers)	52 (8.3)
**How has the outbreak affected your childcare while school was suspended?** ^1^	
My regular childcare has not been affected by the COVID-19 outbreak	792 (81.8)
My spouse/partner or I had to change our work schedule to care for our children ourselves	138 (14.4)
I had difficulty arranging for childcare	26 (2.7)
I had to pay more for childcare	5 (0.5)
**Childrens’ social connectedness to other people and their environment**	888 (91.9)
“Socially connected” Before Pandemic	427 (44.2)
“Socially connected” After Pandemic	

1 Multiple response questions

**Table 3:** Caregivers’ perceived impact of COVID-19 and coping strategies

Variable	n (%)
***Please indicate the extent to which you view the COVID-19 outbreak as having either a positive or negative impact on your life?***	
Extremely negative	393 (40.7)
Moderately negative	292 (30.2)
Somewhat negative	184 (19.0)
No impact	59 (6.1)
Slightly positive	17 (1.8)
Moderately positive	17 (1.8)
Extremely positive	4 (0.4)
**What have been your greatest sources of stress from the COVID-19 outbreak?** ^1^	
Financial concerns	802 (83.0)
Impact on work	763 (79.0)
Access to food	462 (47.8)
Impact on your child	455 (47.1)
Health concerns	263 (27.2)
Impact on family members	203 (21.0)
Social distancing or being quarantined	145 (15.0)
Access to baby supplies (e.g., formula, diapers, wipes)	43 (4.5)
Access to personal care products or household supplies	30 (3.1)
Impact on your community	18 (1.9)
Access to medical care, including mental health care	9 (0.9)
***Please indicate the extent to which you view the COVID-19 outbreak as having either a positive or negative impact on your life?***	
Other (includes loss of relative, accommodation, wear of mask)	14 (1.4)
I am not stressed about the COVID-19 outbreak	53 (5.5)
What have you done to cope with your stress related to the COVID-19 outbreak?^1^	
Meditation and/or mindfulness practices	786 (81.4)
Talking with friends and family (e.g., by phone, text, or video)	444 (46.0)
Increased television watching or other “screen time” activities (e.g., video games, social media)	420 (43.5)
Engaging in more family activities (e.g., games, sports)	106 (11.0)
Eating more often, including snacking	72 (7.5)
Increasing time reading books, or doing activities like puzzles and crosswords	67 (6.9)
Drinking alcohol	22 (2.3)
Volunteer work	7 (0.7)
Talking to my healthcare providers more frequently, including mental healthcare providers (e.g., therapist, psychologist, counselor)	2 (0.2)
Using tobacco (e.g., smoking, vaping)	1 (0.1)
Other (includes sleeping, medication, listening to music)	26 (2.7)
I have not done any of these things to cope with the COVID-19 outbreak	54 (5.6)

1 Multiple response questions

**Table 4:** Caregivers’ reported household food situation during COVID-19

Item	Mean (SD) or %	value
Likert scale “level of worry” about household food situation during the COVID-19**:		
*My household will not have enough food if we have to stay at home and can’t go out at all (due to quarantine or illness)*	5.0 (1.2)	
*Food will become more expensive for my household*	4.8 (1.4)	
*My household will lose so much income that we cannot afford enough food*	4.7 (1.4)	
*There will not be enough food in the marketplace*	4.0 (1.7)	
*The country will not have enough food to feed everyone*	4.0 (1.7)	
*Food will become unsafe or contaminated*	3.6 (1.8)	
**Percentages of caregivers reporting agreement with statements on household food situation**		
**The food the household bought did not last, and there was no money to get more**		
One year before outbreak	19.6	
Since outbreak	55.4	< 0.001
**My household could not afford to eat balanced meals**		
One year before outbreak	17.1	
Since outbreak	48.8	< 0.001
**Household members ate less food than they felt they should because there was not enough money for food**		
One year before outbreak	17.3	
Since outbreak	42.7	< 0.001
**Adults in the household ever cut the size of meals or skipped meals because there was not enough money for food**		
One year before outbreak	15.0	
Since outbreak	41.4	< 0.001
**Adults in the household were ever hungry but did not eat because there wasnot enough money for food**		
One year before outbreak	6.0	
Since outbreak	18.4	< 0.001

** The Likert scale ranged from 1 (not at all worried) to 6 (extremely worried)

## Data Availability

All the data underlying this study is available at https://osf.io/bmv9d/

## Abbreviations

COVID-19: Coronavirus Disease 2019
iLiNS: International Lipid-Based Nutrient Supplement
